# Traditional climate knowledge: a case study in a peasant community of Tlaxcala, Mexico

**DOI:** 10.1186/s13002-016-0105-z

**Published:** 2016-08-18

**Authors:** Alexis D. Rivero-Romero, Ana I. Moreno-Calles, Alejandro Casas, Alicia Castillo, Andrés Camou-Guerrero

**Affiliations:** 1 Escuela Nacional de Estudios Superiores, Unidad Morelia, Universidad Nacional Autónoma de México, Antigua Carretera a Pátzcuaro 8701, 58190 Morelia, Michoacán Mexico; 2 Instituto de Investigaciones en Ecosistemas y Sustentabilidad, Universidad Nacional Autónoma de México, Antigua Carretera a Pátzcuaro 8701, 58190 Morelia, Michoacán Mexico

**Keywords:** Agriculture, Climate prediction, Propitiatory ritual

## Abstract

**Background:**

Traditional climate knowledge is a comprehensive system of insights, experiences and practices used by peasant communities to deal with the uncertainties of climate conditions affecting their livelihood. This knowledge is today as relevant in the Mesoamerican and Andean regions as it is in Europe and Asia. Our research sought to analyze the traditional knowledge about the weather and climate in a rural village of the state of Tlaxcala, Mexico, and its importance in decision-making in agriculture.

**Methods:**

Through 30 interviews and participant observation in the community during 2013, information was gathered about traditional climate and weather indicators and prediction tools, as well as rituals and agronomic and agroforestry strategies. This information allowed for the reconstruction of the community’s agro-festive calendar. Data analysis was carried out with the help of the qualitative analysis software Atlas.ti (version 7).

**Results:**

The socio-ecological importance of traditional knowledge about the climate lies in its ability to forecast local weather conditions and recognize climate variations, so vital to the food security of rural families. Knowledge about climate predictors is exchanged and passed on from generation to generation, contributing to the preservation and promotion of biodiversity. By observing the behavior of 16 animals and 12 plant species (both domestic and wild) as well as seven astronomical indicators, villagers are able to predict rain, dry weather and frosts. However, the continuity of this traditional knowledge in the community under study is now compromised by the little interest in agriculture characteristic of the younger generations, the ensuing abandonment of the countryside, the widespread economic crisis and the disappearance of animal and plant species.

**Conclusions:**

Traditional climate knowledge includes the understanding of weather events and weather changes at different time scales (hours, days, weeks, and seasons). The ability to interpret weather events thanks to the accumulated knowledge about the climate through generations may prove today a relevant tool for improving agricultural practices and dealing with local and global socio-ecological changes.

## Background

Global changes intensify and become increasingly more uncertain, the risks to human life multiply. Agriculture, fishing, and other subsistence activities are currently vulnerable to local climate change impacts [1, 2]; For instance, variations in rainfall regimes have led to a reduction in agricultural production during relatively long periods and increased the probability of failure in short-term cropping [3]. Similarly, the rises in average global temperatures favor the proliferation of weeds and pests at the expense of wanted crops [4].

Studies on the impacts of climate change on water resources, rainfall patterns, biodiversity and agro-biodiversity suggest that changes will be more intense for developing countries [5, 6] where special ecological conditions, rural poverty and hunger due to the unequal distribution of income, land and water, threaten the continuity of small rural production systems [7–9].

To address this situation, a number of political and technological adaptive responses have emerged, of which the most well-known are those proposed by international organizations such as FAO and IPCC [7, 10]. However, little attention has been given to the local knowledge about climate dynamics accumulated by peasants through the centuries, and their reactions to the present state of affairs. Concern about variations in climate dynamics has been present in human cultures since time immemorial [11]. Around the world, local peoples have developed environmental knowledge systems that have allowed them to continuously produce the food necessary for survival under different and varying conditions [12].

Local experiences of climate include detailed observations at different scales in time and space, and are useful as a complement to instrumental climatic data [13]. Traditional Climate Knowledge (TCK) has been constructed through detailed observations of the environment, such as the behavior of animals, changes in the morphology and the physiology of plants, patterns in the formation and properties of clouds, the appearance of the moon and other celestial bodies, and other meteorological phenomena that are useful to climate prediction [14–16].

In agriculture, the anticipation of the arrival of the rainy season and the amount and intensity of rainfall, as well as the occurrence of frosts and drought is of great importance to avoid catastrophes. Traditional climate-prediction skills are diverse, based on multiple signs and sources of information, and they influence the decisions and practices associated with growth cycles, the varieties of crops that should be managed, and other agronomic, forestry and fishing activities [17]. Anticipating or predicting the weather involves not only the observation of nature, but also an interpretation of its elements and processes, necessary to formulate the best guess about future climate conditions [18].

Although the patterns of TCK in the Mesoamerican and the Andean regions are similar to those found in Europe and Asia [19], each socio-ecological context possesses its own characteristics and deserves close scrutiny. Included in the traditional knowledge about the climate are propitiatory rituals associated with religious beliefs and social mores, through which petitions for good times are made to different divinities and gods [20]. Peasants use traditional climate predictors (TCP) to forecast weather events in the next hour, day, week or season by means of an interpretation about climate constructed in the long term [16, 18, 21, 22]. Climate and weather prediction and the propitiatory rituals and beliefs associated to it are the products of many years of careful observation, and experience. This knowledge, passed on from generation to generation, continues to be relevant to the success of productive activities [21].

Studies in Ethnoecology, Ethnoclimatology, Ethnometeorology, Agronomic Sciences and Environmental Anthropology have been documenting the beliefs, insights, knowledge and actions of human societies in relation to climate in the short, medium and long terms [16, 18, 19]. TCK includes the duration and intensity of rainfall, drought, frost, and wind and their influence on farming decisions and in other primary productive activities [21–24].

This study was conducted in El Carmen Tequexquitla, Tlaxcala. It is the first of its kind in the area, although there are some publications concerning the neighboring Puebla-Tlaxcala Valley [16, 20, 22]. Endowed with a semi-arid landscape and a severe climate with a high incidence of autumn frosts [25], the site was selected for its vulnerability to the effects of climate change given the high levels of environmental degradation prevalent in the area, due to deforestation, land use change and the intensification of agricultural practices [26].

The study’s main objective was to analyze the theory and practice of traditional climate prediction in El Carmen Tequexquitla and the way this knowledge is constructed. We documented in particular: i) the existing traditional knowledge about the environmental factors associated to climate, ii) the elements for climate prediction and their relations to agricultural practices, iii) the current state of knowledge about the climate and the factors and processes influencing its transmission, and iv) the influence of traditional meteorological prediction on future agricultural management decisions in the context of climate change.

## Methods

### Study area

**Fig. 1:**
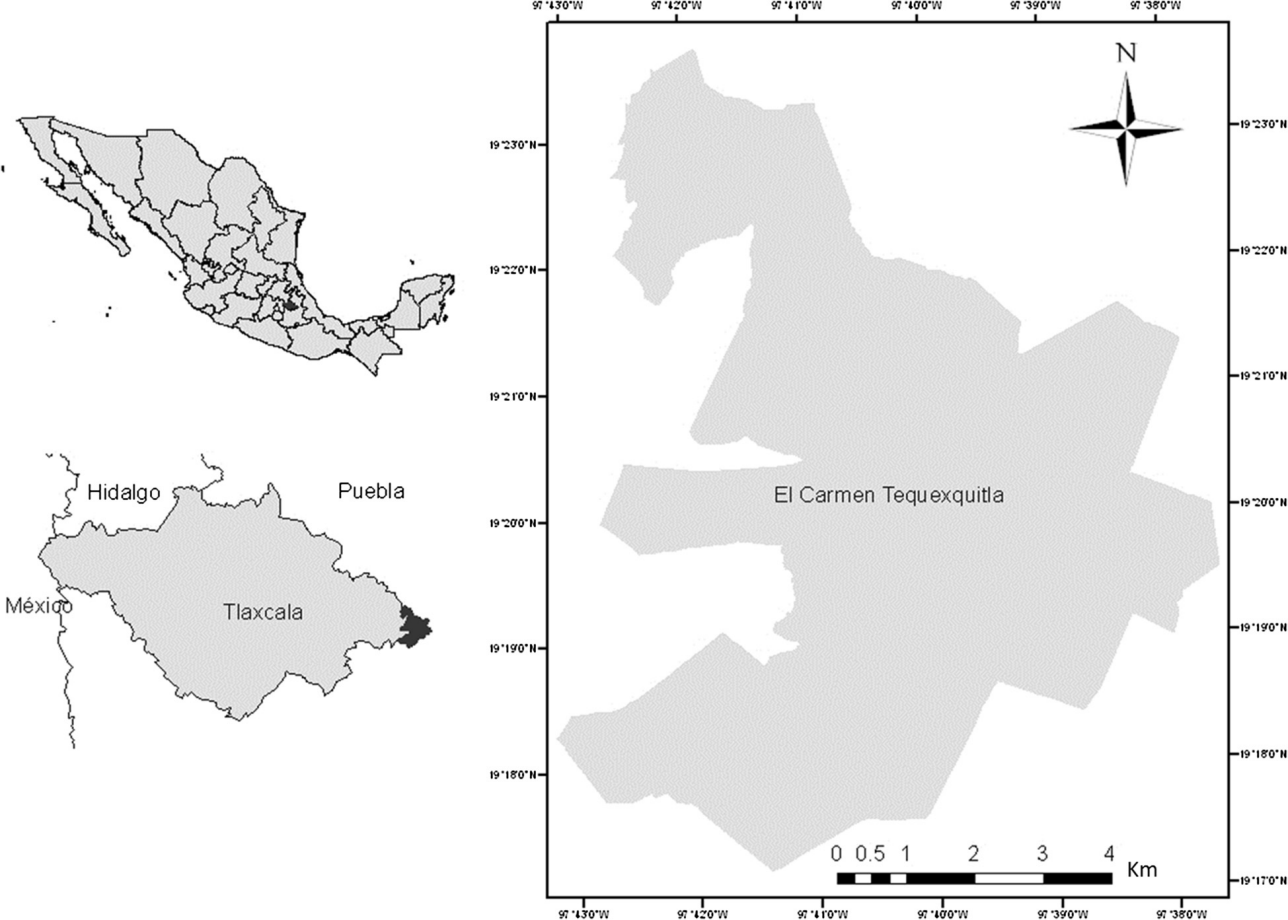
Location of El Carmen Tequexquitla, Tlaxcala, México

**Fig. 2:**
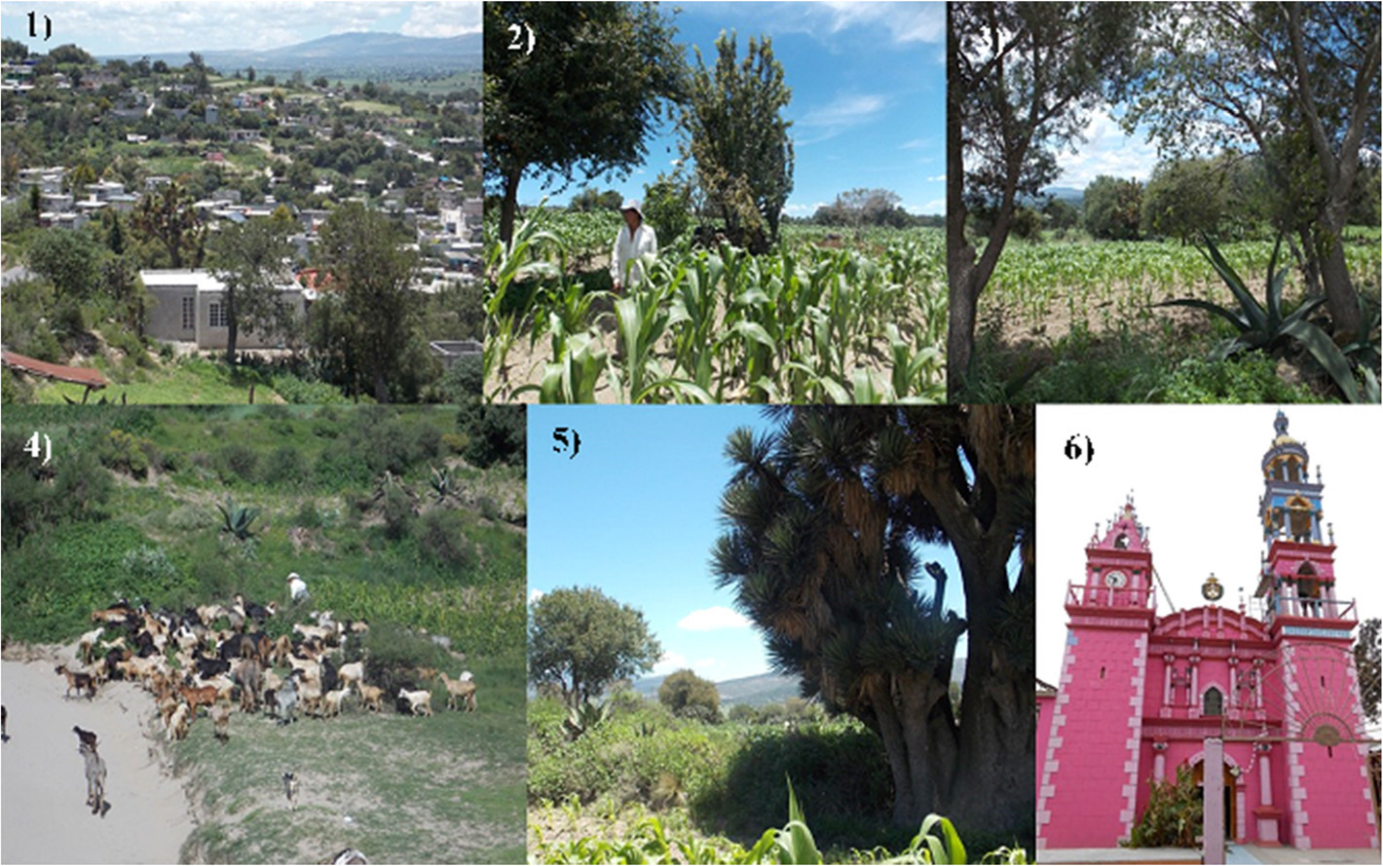
Climate indicators at El Carmen Tequexquitla. 1) Carmen Tequexquitla landscape; 2) traditional agroforestry system; 3) maize cultivation associated with useful species for climate prediction, such as *maguey* and *capulín*; 4) to contain cattle, a cattle herder enlist goats, an animal used as weather predictor; 5) the *izote* dominates the agricultural landscape at El Carmen Tequexquitla, and 6) The church of the “Virgen del Carmen”, a ritual space of great importance to peasants in the community

The municipality of El Carmen Tequexquitla in the state of Tlaxcala spreads over an area of 58.5 km^2^ amid the Balsas and Atoyac river basins on the Mexican Transversal Neovolcanic Axis [27]. It comprises five communities (Guadalupe, Vicente Guerrero, Temalacayuca, La Soledad and Mazatepec) with a total population of 15,368 people [28]. In El Carmen Tequexquitla only 769 people (0.5 %) speak Nahuatl and the rest of the population speaks only Spanish [28]. However, plants, animals and places are named in both Nahuatl and Spanish [29] (Figs. 1 and 2).

The municipality is located between 2,400 and 2,700 masl, with mean annual temperatures ranging from 12 to 16 °C, and mean annual rainfall from 420 to 520 mm [30]. The dominant vegetation is dry scrub, an ecosystem with different kinds of succulent and thorny shrub plants, with some trees and shrubs such as *tlaxcas* and *sabinos* (*Juniperus flaccida* Schltdl. and *Juniperus deppeana* Steud.) and *izotes* (*Yucca* ssp.), and pine forests dominated by pinyon pine (*Pinus cembroides* Zucc.) [31, 32].

### Research design

In preparation for fieldwork, the *ejido* and municipal authorities of El Carmen Tequexquitla were contacted to: i) assess the relevance of our study on the existing local knowledge about the climate; ii) discuss the importance of sharing this knowledge, and iii) seek the support of the authorities on fieldwork logistics and the conduction of the interviews. The commitment was established at these interviews to share the research findings with the community in the form of both a written report and a presentation on the current status of the local knowledge about the climate and the environment, its importance and the threats it presently faces. These actions led to the construction of strong links between the researchers and the local people, whose collaboration with the project helped reinforce in them an attitude of commitment and respect necessary for collaborative learning. In order to document and understand traditional knowledge about the climate, we used a qualitative-interpretive research approach that allowed for the examination of the subjects’ own perspectives about the research topic [33].

### Compilation of data

During the first visits to the community, we sought to identify our initial key subjects, namely those considered ‘experts’ in climatic indicators. A total of 30 semi-structured interviews were conducted with an equal number of persons. All interviews were audio recorded for further analysis. The selection of interviewees followed the “snowball method”, by which an informant or group of key informants lead to other individuals who possess information relevant to the study [34, 35]. Sample size was determined through “theoretical saturation”, which prevents data repetition and ensures its representativeness [36].

Participant observation, on the other hand, allowed us to interact with people while conducting their daily activities. During 2013 we accompanied our informants to their agricultural plots and forest areas to identify climate predictors and to learn about their use and their purpose in agricultural activities. Finally, we carried out a participatory workshop in order to reconstruct the community’s agro-festive calendar. We recorded the agricultural practices and social activities associated to the cycles of maize production. The agro-festive calendar was of great help in identifying the climatic, agricultural and astronomic elements involved in the organization of activities and festivities in an integrated view of natural phenomena, decision-making processes and socio-cultural activities related to agricultural practices. The workshop proceeded with open questions emphasizing the meteorological knowledge possessed by the participants, including: i) the identification of climatic indicators in the local environment; ii) the propitiatory rituals associated to rain and the prevention of frosts; iii) the timing of events and indicators; iv) the factors that influence local knowledge about the climate, and v) the relationship between all of the above [37].

### Data analysis

Qualitative data from interview transcripts and field notes were analyzed by way of an Atlas.ti computer program (version 7), which facilitates data storage and particularly the coding and construction of categories, leading to the identification of the aspects that are relevant to the informants. Through such analysis, it is possible to systematize and interpret the meanings assigned by people to environmental and social phenomena [38].

## Results and discussion

### Traditional climate knowledge

In El Carmen Tequexquitla peasants have observed and experienced different biotic and abiotic phenomena. According to the people interviewed, the most important weather events influencing agriculture in the region are frosts, drought, rain and wind. Frosts are particularly destructive, although other phenomena also affect crops in varying degrees. An opportune prediction of climate variability in the short, medium and long term is therefore crucial to safeguard the food security and economy of rural families. Knowledge about the climate and its influence on agricultural activities has been of vital importance for social and cultural reproduction in rural contexts [23]. Climate predictions lead to a variety of strategies, including propitiatory rituals and changes in agronomic practices [21].

Through their continuous observations and experiences, peasants have learned to identify what they recognize as “signs of nature” in different phenomena associated to weather, and they have used those signs as climate predictors. They know how a particular kind of weather relates to particular changes in the components, phenomena or processes of an ecosystem or the general environment along the agricultural cycle. Through a ‘reading’ of the environmental signs related to climate [19], people are capable of predicting a “good” or a “bad” year (a definition relative to the regularity or irregularity of rains and the occurrence of drought or frosts). Similar information has been published for other communities on the Puebla-Tlaxcala valley [16, 22] and other parts of the world [13, 15, 19, 21, 39].

In El Carmen Tequexquitla, the observation of natural indicators during the first five calendar months (January to May) allows peasants to predict major meteorological trends for the remaining months of the year relevant to the agricultural cycle. This information is complemented with more detailed observations of TCP during the months of crop development. Throughout the agricultural cycle people pay attention to “signals” of possible atypical situations. Such indicators are used for short-term prediction of possible variations in climate from one day to another, and up to one week.

### Inventory of traditional climate predictors

The observation of climate predictors is a useful skill inherited from past generations, and the large number and variety of indicators recognized by the people interviewed in the study attest to its relevance (See Tables 1, 2 and 3). A total of 29 species of plants and animals (domestic and wild) were recorded, in addition to seven astronomical events used as climate predictors (Table 4). With this information we proceeded to build one of the most detailed descriptions of TCP in Mexico [15, 16, 18, 40].

**Table 1:** Vegetal climatic predictors operation

Local (Species) Name	Month Observation	Description
Maguey (*Agave salmiana* Otto ex Salm-Dyck.)	April	When the flowering of this plant is very rich in terms of landscape, a season is expected with good quality showers.
Nopal (*Opuntia* sp. L.)	April-May	The abundance of suckers on the pads of the prickly pear indicates favorable rains for crops.
Azomiate (*Senecio salignus* DC.)	March-April	The early and abundant flowering of this plant indicates good quality of rain during agricultural work.
Escobillo (*Asteraceae* sp. Bercht & Presl.)	March-April	Its abundant flowering, tells them to Peasants temporary quality (regular rainfall) for crops.
Izote (*Yucca* sp. L.)	January-February	The emergence of a large number of “palmos” (inflorescence) indicates good quality of rains, while its incipient flowering indicates a bad time.
Sotol (*Dasylirion* sp.)	January-February	Its abundant flowering is indicative of a temporary evil, little rain.
Maize (*Zea mays* L. Zucc.)	June-August	When the maize plant shows wilted appearance, even when the ground is wet, it is a sign of "bad weather" or the next occurrence of frost.
Nogal (*Juglans regia* L.)	January-February	The early flowering of this tree is indicative of frost or cold weather during the corn growing.
Fennel *(Foeniculum vulgare* Mill.*)*	March-April	The abundant flowering of this plant is a sign of a good harvest.
Peach (*Prunus persica* (L.) Batsch) Capulín (*Prunus capuli* Cav.) Plum (*Prunus domestica* L.)	January	Early flowering of these trees is an indicator of "good time", regular rainfall. On the other hand, if the flowering is delayed, is not abundant or is affected by a cold, this indicates "bad time", irregular rainfall or the appearance of some frost during the growth of the maize.

**Table 2:** Animal climatic predictors operation

Local (Species) Name	Month Observation	Description
Tuza (*Geomys mexicanus* B.)	March	In the planting season when they have already fallen the first rains, if this rodent goes (makes cavities around the maize) in the morning, it means that for that day or the next heavy rains fall. On the contrary if it goes in the evenings, it is an indication that the rain will stop in the following days.
Tecolote (*Strigidae* sp.)	March-April	When this bird sings or "squealing" in the evenings is a bad omen, either in farming or in death or an accident.
Rooster (*Gallusgallus domesticus* L.)	April-August	During the rainy season, when the cock crows after hours (afternoon or evening) it is indicator that you will stop for a while.
Black ant (Formicidae sp.)	March	When black ants begin to line up across the width of a sidewalk or road, it is a sign of coming rain.
Red ant (Formicidae sp.)	March-April	When this insect begins to build mounds in his career, it means that soon fall favorable rains for agriculture.
Coyote (*Canis latrans* Say.)	April-May	The coyote is an animal that can rarely be seen, but through his howl Peasants can predict good or bad weather. When his howl is melodic, it indicates next rains, whereas if the howling is obnoxious or clipped, it will not rain soon or be dry during the growth of the maize.
Maguey butterfly (*Acentrocneme hesperiaris* Walker.)	March-May	When the landscape abound in this kind of small butterflies (brown or red), Peasants interpret the rains will be good quality, that is, on time and in adequate quantity.
Mosquitos (Culicidae spp.)	May	When "rampage" and leave in large groups, it is a sign that heavy rains are coming.
Goat (*Capra aegagrus hircus* L.) Dogs (*Canis lupus familiaris* L.)	April-May	Cheerful and impetuous behavior of these animals it is a sign of good weather (heavy rain or hail frost). A passive and sad behavior indicates cessation of rains and possible occurrence of frost or drought.
Mare (*Equus ferus caballus* L.)	July-August	When horse trembles in the evenings is a sign that a frost is near to fall on the fields.
Swallow (*Hirundo rustica L.)*	April-May	If this bird flies at low altitudes near the planting season, it means that it will rain in the coming days and that the quality of rainfall will be favorable for crops.
Wren (*Troglodytes aedon* V.)	May-July	If you are singing on the trees in the morning, it is an indicator of air streams.
Cuitlacoche (*Taxostoma curvirostre* S.)	May-July	If your song is by the morning is omen to air or frost.
Lili (Bird)		When he sings in the evenings, it's a sign that fall frost in the next day or the same day of the signal.
Pashira (Bird)		If this bird sings insistently by the evening it is an omen of "bad weather", moreover if sings early is a sign of "good time".
Chupepe (Bird)		It is a sign of rain when this insect (like the Beetle) is out of the nest, the roadside or in the maize field.

**Table 3:** Abiotic climatic predictors operation

Local Name	Month Observation	Description
“Malinche nevada” (Local snowy volcano)	June-August	When the snows Malinche in temperate seasons, is a sign that in the coming days will drop a frost.
Lightnings	April-May	This event has interpreted two ways. On the one hand if lightnings are about Popocatepetl (northwest volcano), it is indicative of continuous rain or forthcoming. And secondly, if the lightning appear on the Pico de Orizaba (northeast volcano) is a sign that will be delayed or dry abundance.
Moon	June-July	The moon has two functions as environmental predictor climate, abundant rainfall coloring indicates if it is completely white and beige tones if you have a dry season. If the moon is tilted south rain and indicates whether sloping north is dry.
“Serpiente de agua” Watersnake	May	It is called "water snake" a cloud that appears at ground level, leaving moisture in the soil and indicates the arrival of the rains.
Red sky	August-September	Red sky at sunset is a sign cold, that is, the appearance of frost.
Lagoon	July-August	When the lagoon increases its volume before the rainy season, this will mean that quality and favorable for the crops

**Table 4:** Taxa used for climate prediction

Climate Environmental Predictor	Species or type number
Plants	12
Birds	7
Mammals	5
Insects	4
Reptiles	1
Abiotic events	7
Total	36

### Plant predictors

Wild and cultivated plants are used for climate prediction. Their observation takes place mostly at the time of agricultural activities, from January to August (Table 1). These plants are commonly found inside or around the agricultural plots, and they are part of the region’s agroforestry systems. A total of 12 plant species were mentioned during the interviews, the most common and significant among them being the *izote* (*Yucca* sp.) and *sotol* (*Dasylirion* sp.). In these two species, blooming is the climate predictor that most of the interviewees identified. The emergence in *izote* of a large number of *palmos* (flowering shoots) indicates the arrival of abundant rains, whereas its incipient flowering indicates unfavorable conditions. As for the *sotol*, its abundant flowering is indicative of hard times with scarce rain. The early blooming of species like peach, plum and *capulín* (*Prunus serotina* Ehrh.) indicates the arrival of “good weather” (*buen tiempo*) with regular rainfall. Conversely, a late blooming indicates “bad weather” (*mal tiempo*), irregular rainfall or the appearance of frost during the growth cycle of maize. Other indicators are described in Table 1.

### Animal predictors

Animal-based predictors were the “signs” most frequently mentioned in relation to weather conditions. A total of 16 different species were mentioned, of which the most relevant were the gopher (*Geomys mexicanus* B.) and birds such as wren (*Troglodytes aedon* V.), thrasher (*Toxostoma curvirostre* S.), swallow (*Hirundo rustica* L.) and *pashira*, an unidentified species (Table 2). As in the case of plant species, wild and domesticated animals are used for predicting variations in the weather. They are commonly found near the homes, which facilitates their continuous observation and the inventory of their features. For instance, the trembling of a horse (*Equus ferus caballus* L.) in the evenings is a sign that a frost is about to fall. Similarly, when a goat (*Capra aegagrus hircus* L.) shows a cheerful and impetuous behavior, it is signaling “good weather” (i.e., sufficient rain for the growth of crops), while passive or sad behavior in both these animals indicate the cessation of rains and the possible occurrence of frosts or drought (Table 2).

### Astronomical predictors

Predictors of this kind relate directly to changes in atmospheric conditions affecting the appearance of the sky, the water bodies and streams, and the type, intensity and direction of the winds. Such changes are clearly visible, and local people consider them as clear signs. The moon has two functions as climate predictor: When completely white, abundant rainfall can be expected, while beige tones are indicative of a dry season. If the moon is tilted towards the south, there will be rain; when sloping north, it indicates dry weather (Table 3). According to the people interviewed, the moon cycles, locally called *dietas de la luna* define good or bad harvests. During full moon the incidence of pests such as the maize weevil (*Sitophilus zeamais* Motschlsky) decreases, and plants are provided with the necessary strength to grow. Thus, along with climate prediction, an astronomical event indicates the time of sowing. In connection with this, one woman peasant mentioned that: *“You see, the seed is not planted during tender moon because it does not germinate and becomes rot, and if one will were to also harvest during the tender moon the seeds will not mature”* 2013). There are other important predictors like *serpiente de agua* (water snake), a cloud that appears at ground level leaving moisture in the soil, which indicates the arrival of rains (Table 3).

### Traditional worldview of the climate

**Fig. 3:**
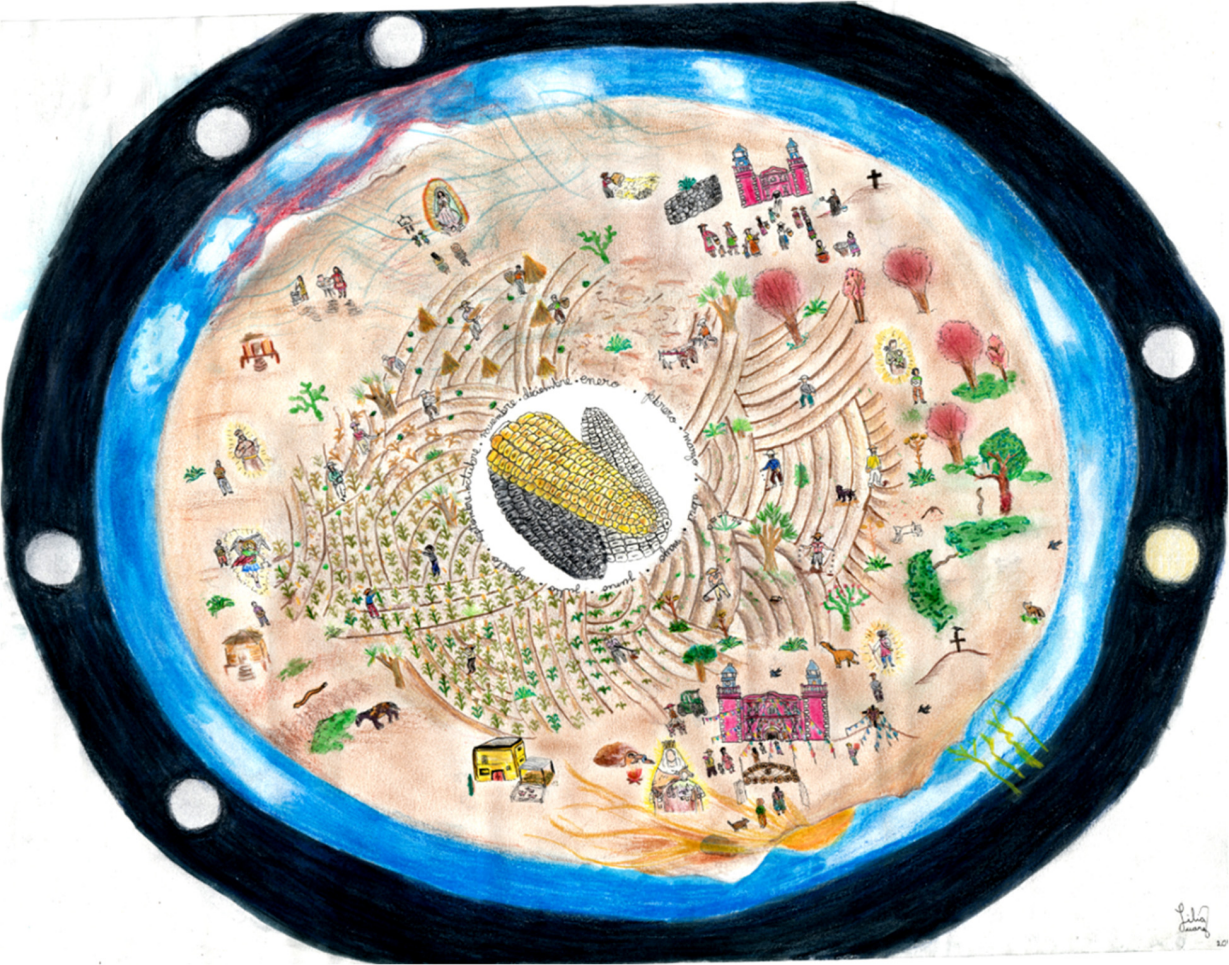
Agro-festive calendar of El Carmen Tequexquitla. Maize is placed at the center. The second section shows the agricultural activities (fallow, planting, germination control and harvest). Environmental predictors appear at their corresponding time of year. Finally, the outer section of the calendar represents the sky, where atmospheric dynamics takes place, and the outer space, where the lunar cycle proceeds

Along with TCP, in El Carmen Tequexquitla people use intangible resources to deal with uncertain climatic events, including those belonging to the set of beliefs or conceptions about the world and the elements that are part of it, i.e., their *worldview* or *Kosmos* [41]. Every peasant family performs some rituals that serve as a link between productive activities and their spirituality and religious inclinations [18, 20] (Fig. 3).

In the community studied, the climate is associated with a large number of saints and ceremonies whose role is to intercede for “good weather”. For example, rainfall on the day of San José (19 March) beacons a good harvest, while unstable weather on the day of San Miguel (29 September) is a fateful sign (Fig. 3). Traditional climate knowledge is a complex set of beliefs, experience and practices that cannot be understood separately, but must be considered as an integral whole within a particular space, time and human context. This is how in El Carmen Tequexquitla people practice and recreate their agricultural wisdom and knowledge.

### Socio-ecological relevance of TCK

For the local people interviewed, knowing how the climate works was very important in the context of rural life, as it allows them to: i) forecast local climate variations, ii) enhance the food security of their families, iii) establish an inter-generational dialogue, and iv) maintain bio-cultural diversity.

### Local weather forecast

Our research results illustrate the enormous importance TCP has for agriculture and other primary productive activities, not only for the intrinsic value of this wide and deep knowledge about meteorological signs, but because this knowledge is part of traditional ecological knowledge up keeping, renewed from one generation to another. For older peasants in particular, who depend entirely on seasonal agriculture, knowing about climate variations and how to predict them means to be a “good peasant” or an “expert peasant”, with a greater probability of success in agricultural activities.

### Inter-generational dialogue

The people of El Carmen Tequexquitla are aware of the importance of preserving TCK and their time-honored predicting methods, including the understanding of climatic and meteorological indicators. This knowledge is preserved through an inter-generational dialogue that strengthens both family and social ties and cooperation among peasants. However, as in many other areas of rural Mexico, today the communities of El Carmen Tequexquitla find it difficult to make a living out of farming and have decided to look for opportunities elsewhere.

### Food security in El Carmen Tequexquitla

TCP for the prevention of possible negative outcomes in agriculture, along with other activities that ensure a good food harvest or mitigate the effect of natural disasters on crops, conform a set of strategies to deal with uncertainty, and play a key role in guaranteeing the food security of the community.

### Socio-ecological resilience and the preservation of bio-cultural diversity

The knowledge and use of TCP has also favored the conservation of ecosystems and the promotion of biodiversity. For weather prediction purposes, preserving plant and animal species and the spaces where they live is essential. Adult and elderly peasants believe that the promotion of this knowledge among young peasants will contribute to the conservation of such diversity. Naturally, the more accurate predictions are, the more interest will they elicit in young farmers in preserving the knowledge about the species of predictors and meteorological events, thereby contributing to bio-cultural conservation. Furthermore, in order to reduce the risk of major losses in food production in the future, young peasants will in turn develop strategies for the prevention, mitigation and adaptation to possible climatic variations. Socio-ecological resilience (i.e., the ability to cope with environmental contingencies and recover from them through different practices) depends on traditional meteorological knowledge and the risk-prevention practices generated from them [16, 42].

### Processes that influence traditional climate knowledge in El Carmen Tequexquitla

People’s perception about the loss of traditional knowledge (or what in El Carmen Tequexquitla is called “the knowledge of the past”) varies considerably according to the age of the peasants, the time they devote to agriculture, their origin and their contact with city life. However, everybody agrees that “things are not as they used to be” and that agricultural practices are in danger of disappearing along with those who practice them. What follows is a description of the general factors believed by peasants to directly affect the current state of local knowledge.

### Severe weather changes

According to peasants, this is one of the factors that dissuade them from using the accumulated knowledge about the climate and the environment, including their associated predictors, as the weather seems to change more dramatically each year and prediction becomes even more difficult to use and increasingly less reliable for making decisions about agricultural activities.

### Young people’s disinterest in agriculture and the abandonment of the countryside

Young people of El Carmen Tequexquitla have begun to emigrate to a variety of places, from the nearest cities like Tlaxcala to places abroad in search of better education or work. This situation, along with an existing lack of interest in agricultural activities, has led to the abandonment of productive areas in recent times. The physical exertion associated to agricultural tasks along with the falling prices of products (maize, wheat, barley) in the market, have made agriculture an altogether ungenerous economic activity and an increasingly unattractive occupation for the young.

### Widespread economic crisis

In the communities under study, peasants point to the national economic crisis as determinant to the current abandonment of agriculture and the loss of knowledge associated with it. They see the rural sector as forgotten and neglected by the federal and state governments, as well as other relevant decision makers. The high cost of agricultural inputs, the fall in labor wages and produce prices and the lack of government support are, in their opinion, some of the factors that trigger agricultural abandonment. This situation is in tune with the findings of a study about the rural sector in Latin America [27] where, according to the authors, between 1990 and 2005 poverty levels ranged around 35 million people.

### Disappearance of species used for weather prediction

Peasants recognize that many of the species used for weather prediction are disappearing, including swallows and other migratory birds. These species have been affected by both hunting and the disappearance of their habitats, and many of them have been forced to alter their migration routes even as they see their populations dwindle.

## Conclusions

The findings in this study lead us to argue that traditional knowledge systems about the climate and the environment (as well as other knowledge systems and practices of peasants and native peoples) provide their users with the necessary tools to adapt to the peculiarities of a given environment.

Traditional knowledge about the climate remains an almost unexplored area of study in Mexico, despite their bearing on the development and dynamics of its original peoples. This study demonstrates the importance this knowledge has for those who engage in agricultural activities in the municipality under study, and probably beyond. TCK and TCP are part of the “toolbox” that peasants possess to carry out agricultural practices, and further in-depth studies could open up new spaces for decision-making in view of the ongoing processes of climate change and weather variability at the local level. A list of species and their attributes could be composed and applied to this very purpose, as well as to other conservation strategies.

TCK is part of the immaterial wealth of peasants and original peoples of Mexico, which, despite its enormous importance, is in danger of extinction due to a number of processes associated with the modernization of the rural world and the little value generally accorded to rural traditional knowledge. Such trend can be broken and even reversed, provided steps are taken to recover traditional knowledge and strengthen rural peoples’ dignity and their rights to maintain their livelihoods.

## Abbreviations

TCP: Traditional Climate Predictors
TCK: Traditional Climate Knowledge
FAO: Food and Agriculture Organization of the United Nations
IPCC: Intergovernmental Panel on Climate Change
CEPAL: Economic Commission for Latin America and the Caribbean
